# Correction to: Darbepoetin-α increases the blood volume flow in transplanted pancreatic islets in mice

**DOI:** 10.1007/s00592-021-01771-1

**Published:** 2021-07-28

**Authors:** Maximilian M. Menger, Lisa Nalbach, Selina Wrublewsky, Matthias Glanemann, Yuan Gu, Matthias W. Laschke, Michael D. Menger, Emmanuel Ampofo

**Affiliations:** 1 Institute for Clinical and Experimental Surgery, Saarland University, 66421 Homburg, Saar Germany; 2 Department for General, Visceral, Vascular and Pediatric Surgery, Saarland University, 66421 Homburg, Saar Germany

## Correction to: Acta Diabetologica (2020) 57:1009–1018 https://doi.org/10.1007/s00592-020-01512-w

The article “Darbepoetin‑α increases the blood volume flow in transplanted pancreatic islets in mice”, written by Maximilian M. Menger, Lisa Nalbach, Selina Wrublewsky, Matthias Glanemann, Yuan Gu, Matthias W. Laschke, Michael D. Menger and Emmanuel Ampofo, was originally published Online First without Open Access. After publication in volume 57, issue 8, page 1009–1018 the author decided to opt for Open Choice and to make the article an Open Access publication. Therefore, the copyright of the article has been changed to © The Author(s) 2021 and the article is forthwith distributed under the terms of the Creative Commons Attribution 4.0 International License, which permits use, sharing, adaptation, distribution and reproduction in any medium or format, as long as you give appropriate credit to the original author(s) and the source, provide a link to the Creative Commons licence, and indicate if changes were made. The images or other third party material in this article are included in the article’s Creative Commons licence, unless indicated otherwise in a credit line to the material. If material is not included in the article’s Creative Commons licence and your intended use is not permitted by statutory regulation or exceeds the permitted use, you will need to obtain permission directly from the copyright holder. To view a copy of this licence, visit http://creativecommons.org/licenses/by/4.0/. Open access funding enabled and organized by Projekt DEAL.

The original article has been corrected.

